# Innovative Immobilization of *Aspergillus aculeatus* OI4C2 Fructosyltransferase on MANAE-Agarose Cross-Linked with Genipin for Enhanced Production of Fructooligosaccharides

**DOI:** 10.1021/acs.jafc.5c13809

**Published:** 2026-02-23

**Authors:** Tatiane Sayuri Inagaki, Diandra de Andrades, Gabriela Souza Alves Picciarelli, Maria de Lourdes Teixeira de Moraes Polizeli, Douglas Poleto de Oliveira, Valdemiro Pereira de Carvalho Junior, Paula Daniela Helfenstein Rother, Mariana Fensterseifer Fabricio, Marco Antônio Záchia Ayub, Plinho Francisco Hertz, Marina Kimiko Kadowaki

**Affiliations:** † Center of Medical Sciences and Pharmaceutical, Western Paraná State University, Rua Universitária 2069, ZC 85819-110 Cascavel, PR, Brazil; ‡ Department of Biology, Faculty of Philosophy, Sciences and Letters of Ribeirão Preto, 54539 University of São Paulo, 14040-901 Ribeirão Preto, SP, Brazil; § Department of Chemistry and Biochemistry, Faculty of Science and Technology, Júlio de Mesquita Filho State University, 19060-900 Presidente Prudente, SP, Brazil; ∥ Biotechnology, Bioprocess, and Biocatalysis Group, Food Science and Technology Institute, 28124 Federal University of Rio Grande do Sul, Av. Bento Gonçalves 9500, PO Box 15090, ZC 91501-970 Porto Alegre, RS, Brazil; ⊥ Enzymology Laboratory, Food Science and Technology Institute, Federal University of Rio Grande do Sul, 9500 Bento Gonçalves Ave, P.O. Box 15090, ZC 91501-970 Porto Alegre, RS, Brazil

**Keywords:** immobilization, fungal enzyme, fructooligosaccharides, prebiotic

## Abstract

This study evaluated the use of genipin in monoamino-*N*-aminoethyl (MANAE)-agarose in the immobilization of fructosyltransferase (FTase) from *Aspergillus aculeatus* OI4C2, focusing on the stability and catalytic efficiency for the production of fructooligosaccharides (FOS) from sucrose. The immobilization of FTase on genipin-cross-linked MANAE-agarose achieved a 98.53% yield, 76.71% activity recovery, and 77.86% efficiency, and it resulted in the synthesis of predominantly short-chain fructooligosaccharides. Comparatively, the maximum amounts of FOS obtained were 73.44 g L^–1^ for the free enzyme and 97.37 g L^–1^ for the immobilized enzyme. Immobilization improved the thermal stability at 40–60 °C and increased the enzyme stability under acidic pH conditions (4.5–6.5) for up to 72 h. The operational stability of the immobilized enzyme retained 50% of its initial activity after 12 reuse cycles. These results indicate that *A. aculeatus* FTase immobilized on MANAE-agarose–genipin is a promising biocatalyst for large-scale FOS production, with prospects in the food, pharmaceutical, and nutraceutical industries.

## Graphical abstract

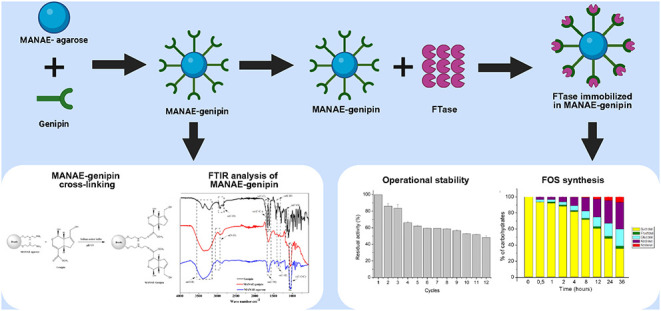

## 1 Introduction

Fructosyltransferase (FTase, E.C. 2.4.1.9) and some β-fructofuranosidase (FFase, E.C. 3.2.1.26)^1^ catalyze the hydrolysis of the β-1,2 linkage in sucrose and transfer the fructosyl moiety to another sucrose molecule, generating fructooligosaccharides (FOS), predominantly 1-kestose (GF2), nystose (GF3), and fructosylnystose (GF4).^2^ These compounds are particularly valued due to their selective fermentability by beneficial gut microorganisms and their associated health-promoting effects.^3^

FTases are frequently produced by filamentous fungi, which represent the main biological source of industrially relevant enzymes due to their high secretion capacity and suitability for large-scale fermentation. Species belonging to the genera *Aspergillus*, *Aureobasidium*, and *Penicillium* are among the most commonly reported FTase producers, with *Aspergillus oryzae*,^4^ *Aspergillus niger*,^5^ *Aspergillus awamori*,^6^ and *Aureobasidium pullulans* ^7^ being extensively exploited at the industrial level.^8^

FOS are recognized as functional foods or prebiotics, as they are resistant to hydrolysis by human digestive enzymes due to β-glycosidic linkages, reaching the colon intact. They play a crucial role in improving gastrointestinal health by selectively promoting the growth of intestinal microbiota, particularly *Bifidobacterium* and *Lactobacillus* species.^9,10^ FOS derived from sucrose also have functional properties such as low-calorie sweeteners that are safe for patients with diabetes.^11^ Their widespread use in the food and pharmaceutical industries is further supported by their noncarcinogenicity, nondigestibility, and potential to reduce blood triglycerides and total cholesterol.^11,12^

The most commonly employed method for large-scale FOS production is the acid hydrolysis of inulin; however, this process suffers from poor selectivity, limited control over the degree of polymerization, the formation of undesirable byproducts, and the generation of chemical waste with potential environmental impact.^13^ Although the enzymatic production of FOS from sucrose is economically advantageous due to the use of a low-cost substrate, its industrial application remains limited by insufficient enzyme stability under variations in pH, temperature, and pressure, as well as by challenges associated with enzyme recovery and reuse.^14^ Enzyme immobilization has emerged as an effective strategy to overcome these limitations, as it enhances enzymatic stability, enables reuse in continuous systems, and can consequently reduce operational costs.^15^ Furthermore, the use of immobilized enzymes represents a more sustainable and environmentally friendly alternative for FOS production, promoting greater process efficiency. Among immobilization approaches, the use of natural cross-linkers has gained increasing attention. Genipin, a biocompatible compound extracted from *Genipa americana* L., has been proposed as an eco-friendly alternative to conventional cross-linkers, such as glutaraldehyde, due to its significantly lower cytotoxicity and environmental impact.^16^ To the best of our knowledge, the application of genipin as a cross-linking agent for monoamino-*N*-aminoethyl (MANAE)-agarose supports in FTase immobilization has not yet been reported, highlighting the novelty and relevance of the present study. In this context, the objective of this study was to immobilize *Aspergillus aculeatus* OI4C2 FTase on MANAE-agarose using genipin as a cross-linker and to evaluate the biochemical properties, operational stability, and efficiency of the immobilized enzyme in the production of FOS from sucrose.

## 2 Materials and Methods

### 2.1 Materials

The analytical-grade standards for HPLC analyses (glucose, fructose, sucrose, kestose, nystose), substrates, and chemical reagents (epichlorohydrin and ethylenediamine) were purchased from Sigma-Aldrich (St. Louis, MO). DEAE-Sephadex, Sephadex G-75, and Coomassie Brilliant Blue R-250 were obtained from Sigma-Aldrich (Brazil). The agarose 6 BCL for immobilization support production was acquired from GE Healthcare Biosciences (Uppsala, Sweden). The glass chromatographic columns were purchased from RBRVIDROS (Brazil). All chemical reagents used in the experiments were of analytical grade.

### 2.2 Microorganism

*A. aculeatus* OI4C2 was isolated from the decomposing material at the Bela Vista Biological Refuge, located in Foz do Iguaçu, Paraná, Brazil, (25°26′37.27″S, 54°33′12.08″W). Species-level taxonomic identification was carried by analyzing the internal transcribed spacer (ITS) regions of rDNA amplified by PCR, following the procedure according to White et al.^17^ The sequence is available in the National Center for Biotechnology Information GenBank under accession number KM382062. For maintenance, the fungal strain was cultivated on potato dextrose agar (PDA) slants prepared in test tubes at 28 °C and subsequently preserved at 4 °C.

### 2.3 Culture Conditions

The fungus was cultivated in 25 mL of Czapek liquid medium with 1.5% soybean meal as the carbon source, contained in Erlenmeyer flasks. The cultivation conditions and the choice of soybean meal were defined based on previous studies.^18^ Inoculation was performed with 1 mL of a spore suspension (2.0 × 105 spores/mL), followed by incubation at 28 °C under stationary conditions for 6 days. The cultures were filtered under vacuum using a Büchner funnel to obtain mycelia, which were washed with water, frozen for at least 2 h, ground with treated sand, resuspended in deionized water, and centrifuged (4000 rpm, 10 min). The clear supernatant obtained was dialyzed against deionized water for 18 h and used for enzymatic activity assays.

### 2.4 Determination of Enzymatic Activity and Protein Concentration

The FTase assay consisted of a mixture of enzyme properly diluted with sucrose at 60% (w/v) in 50 mM sodium acetate buffer (pH 5.5) and incubated at 55 °C, performed according to the methodology adapted from Lateef et al.^19^ The enzymatic reaction was interrupted at specific intervals and boiled at 100 °C for 10 min. The amount of glucose released as a result of FTase activity was quantified according to the methodology of the glucose oxidase–peroxidase kit (Liquiform, Labtest) and measured by absorbance at 505 nm. One unit of enzyme activity was defined as the amount of FTase required to release 1 μmol of glucose per minute under the assay conditions. Protein quantification followed the Bradford^20^ method using BSA (bovine serum albumin) as the standard.

### 2.5 FTase Purification

FTase was purified by centrifugation and dialysis of the intracellular crude extract. A 30 mL sample was pre-equilibrated with 20 mM Tris-HCl buffer (pH 7.2) and then loaded onto an ion-exchange column (DEAE-Sephadex; 2 cm × 0.2 m) equilibrated with the same buffer. Unretained proteins were removed from the column using the same buffer and adsorbed FTase was eluted using NaCl gradient (50–1000 mM) in the same buffer, and fractions were collected into tubes at a flow rate of 4 mL/min. Fractions were assayed for enzymatic activity and protein content. The fractions exhibiting FTase activity were pooled and dialyzed against deionized water for 18 h at 4 °C. Subsequently, the sample was concentrated by lyophilization until reaching a final volume of 500 μL and purified by a gel filtration column (Sephadex G-75; 2 cm × 70 cm) eluted with 20 mM sodium acetate buffer (pH 5.5), and fractions were collected at a flow rate of 2 mL per tube. The purified FTase was kept at −20 °C for subsequent enzymatic immobilization and FOS production.

### 2.6 SDS-PAGE and Zymogram

A 10% acrylamide gel was prepared according to the Laemmli^21^ method to perform SDS-PAGE electrophoresis using the Thermo Scientific PageRuler Prestained Protein Ladder (10–250 kDa). The gel was divided into two portions: one-half was used for Coomassie Brilliant Blue R-250 to staining to analyze protein homogeneity and estimate the molecular weight, and the other half was used for enzyme activity analysis (zymogram) according to Rehm et al.,^22^ with minor modifications. The activity assay employed 0.3 M sucrose in 0.05 M sodium acetate buffer (pH 5.5) as the substrate.

### 2.7 Preparation and Immobilization of FTase on MANAE-Agarose Cross-Linked with Genipin

Genipin was obtained through extraction from *G. americana* L. following the Bellé et al. method.^23^ Glyoxyl was obtained in accordance with Guisán^24^ and subsequently utilized to produce monoamino-*N*-aminoethyl (MANAE) agarose by the Fernandez-Lafuente et al. method.^25^ This support was incubated at 25 °C for 20 h after being cross-linked with 1.5 mg mL^–1^ genipin dissolved in 0.05 M sodium phosphate buffer (pH 7.0).^26,27^ Three pH values (5.0, 7.0, and 9.0) were previously tested, and only pH 7.0 maintained bead integrity. The supports were subsequently rinsed with deionized water and used for enzyme immobilization. Purified FTase was loaded at 5 μg/g of MANAE–genipin support in 0.05 M sodium acetate buffer (pH 5.5) and incubated under gentle agitation (30 rpm, roller homogenizer) at 25 °C for 16 h. The enzyme immobilization process was monitored at specific times by measuring the residual free enzyme activity in the supernatant using Miller’s method,^28^ based on the quantification of reducing sugars (FOS) released, which directly reflects the catalytic activity of the nonimmobilized FTase. The parameters such as immobilization efficiency (EI), immobilization yield (YI), and recovered activity (AR) were calculated as described by Sheldon and van Pelt.^29^

#### 2.7.1 Analysis of Support by Fourier Transform Infrared Spectroscopy (FTIR)

The functional groups of the agarose and MANAE-agarose cross-linked with genipin support were identified by FTIR using a PerkinElmer Frontier spectrophotometer equipped with a diamond ATR module (PerkinElmer, Waltham, MA). Infrared absorption spectra were recorded in the range 200–4.000 cm^–1^ with a resolution of ±1 cm^–1^ and 120 scans.

#### 2.7.2 Biochemical Characterization of Free and Immobilized FTase

The optimum temperature for free and immobilized FTase was determined by performing enzymatic reactions at 20–80 °C. The thermal stability was assessed by measuring the remaining activity after keeping the enzymes at temperatures between 40 and 70 °C for up to 24 h.

The optimum pH was determined by performing reactions in 100 mM buffer systems: citric acid/sodium citrate (pH 3.0–6.0), sodium acetate (pH 4.0–5.5), and sodium phosphate (pH 6.5–8.0), and incubating at 55 °C for 30 min. To evaluate pH stability, the residual activity was measured after incubation in the respective buffers for up to 72 h.

Michaelis constant (*K*_M_) and maximum velocity (*V*_max_) of free and immobilized FTase were determined using sucrose concentrations ranging from 0.05 to 2.0 M. Kinetic parameter values were calculated from a Lineweaver–Burk plot generated with Origin 6.0 software.

#### 2.7.3 Operational Stability of MANAE-Agarose Cross-Linked with Genipin-FTase

The immobilized derivative was incubated with 1.5 M sucrose in 0.05 M sodium acetate buffer at 65 °C and 500 rpm for 1 h. The mixture was then centrifuged at 3000 rpm to separate the reaction product from the immobilized enzyme, which was reused in subsequent cycles. After each cycle, the derivative was washed and centrifuged, and the supernatant was discarded three times using the same reaction buffer to ensure that residues from the previous cycle did not interfere with the next one. The activity of the first reaction cycle was defined as 100%, and the activities of subsequent cycles were expressed as residual activity (%).

#### 2.7.4 FOS Production

FOS production consisted of a mixture of 500 μL of free enzyme and 500 μL of 1.5 M sucrose in 0.05 M sodium acetate buffer (pH 5.5). For the immobilized enzyme, 50 mg of the derivative was resuspended in 500 μL of the same buffer. The reactions were incubated at 55 °C with shaking, and samples were withdrawn at 0, 0.5, 1, 2, 4, 8, 12, 24, and 36 h and stopped in a boiling bath at 100 °C for 5 min. The reaction products obtained from transfructosylation were quantified by an HPLC system (Shimadzu, Tokyo, Japan) equipped with a refractive index (RI) detector and an Amidex HPX-87C column (30 cm × 0.78 cm). The column temperature was set at 85 °C, and ultrapure water was applied as the mobile phase with a flow rate of 600 μL/min. Quantifications were performed using an injection volume of 20 μL, with detection by refractive index (RI) set to a sensitivity of 1000 μRIU/V. Prior to sample analysis, a full calibration curve was prepared and injected; however, the standards were not run before each analytical batch. A calcium-form guard column (Ca^2+^), compatible with Aminex calcium-form analytical columns (pH range 5–9; 2.3 mm × 4.6 mm), was installed upstream to protect the analytical column. Saccharide concentrations (sucrose, glucose, fructose, kestose, and nystose) were determined by interpolation of calibration curves generated using external standards for glucose, fructose, and fructooligosaccharides (kestose and nystose).

### 2.8 Reproducibility and Statistical Analysis

All experimental assays were conducted in triplicate. Data are presented as mean ± standard deviation, and the replicates were used to assess the reproducibility of the experiments.

## 3 Results and Discussion

### 3.1 FTase Purification

The mycelial FTase from *A. aculeatus* OI4C2 was purified 5.6-fold through two chromatographic steps (Figures S1 and S2), with a recovery yield of 38.8% and a specific activity of 40,371.7 U mg^–1^ (Table 1). This specific activity was substantially higher than those reported for other *Aspergillus* FTases including *Aspergillus flavus* (170 U mg^–1^),^30^ *Aspergillus terreus* (1985 U mg^–1^),^31^ and *A. niger* (7047 U mg^–1^).^32^ Enzyme homogeneity was confirmed by SDS-PAGE and zymogram analyses, which showed a single protein band at approximately 110 kDa that coincided with the enzymatic activity band observed in the zymogram (Figure 1). A comparable molecular mass has been previously reported for AcFT2 (100 kDa) from *A. aculeatus*.^33^ However, FTases from other *Aspergillus* species have been described to have both higher and lower molecular weights, such as 135 kDa from *A. aculeatus*,^34^ 50 kDa from *A. oryzae*, and 75 kDa from *A. niger*.^35^

**Figure 1:**
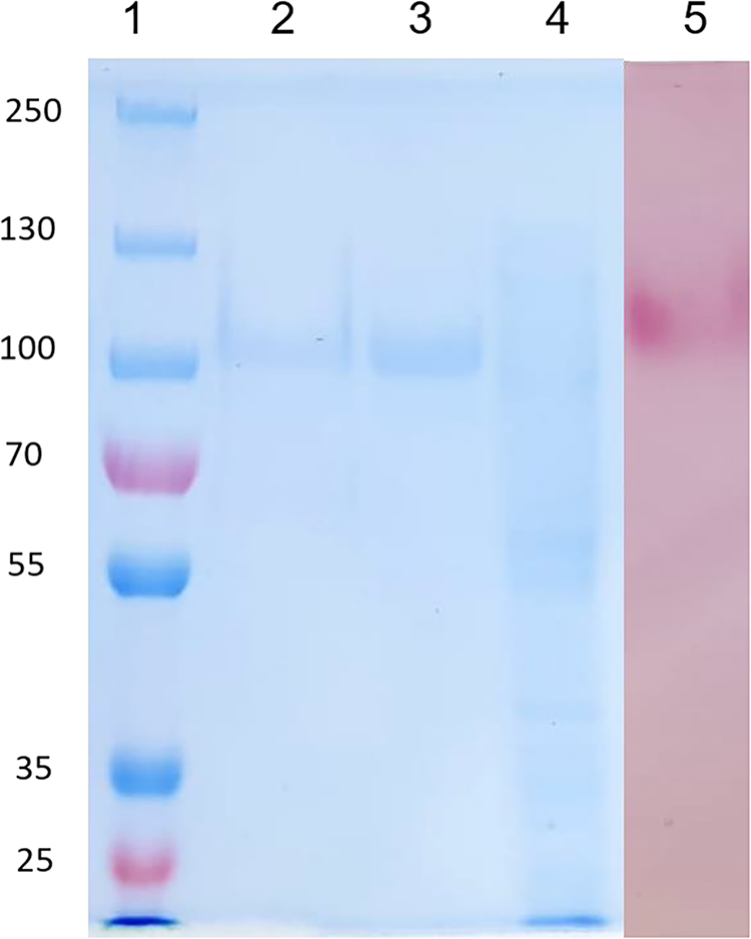
SDS-PAGE electrophoresis and zymogram. Line 1: Thermo Scientific PageRuler Prestained Protein Ladder (10–250 kDa); Line 2: 18 μg of protein sample after Sephadex G-75; Line 3: 24 μg of protein sample after DEAE-Sepharose; Line 4: 28 μg of crude extract; and Line 5: zymogram of purified FTase.

**Table 1:** Summary of Purification of *A. aculeatus* OI4C2 FTase

step	total activity (U)	total protein (mg)	specific activity (U mg^–1^)	purification (factor)	recovery (%)
crude extract	104,775.6	14.7	7127.6	1	100
DEAE-Sepharose	50,100.7	3.8	13,087.9	1.8	47.8
Sephadex G-75	40,694.7	1.0	40,371.7	5.6	38.8

### 3.2 Characterization of the MANAE-Agarose Cross-Linked with Genipin Support

#### 3.2.1 Structural Characterization of the MANAE Support by Fourier Transform Infrared Spectroscopy (FTIR)

Vibrational spectra of the genipin-based agarose support are shown in Figure 2a to confirm the functional groups obtained from the functionalization of agarose with genipin to produce the MANAE-agarose cross-linked with the genipin support. In genipin, the hydroxyl group vibration ν­(O–H) related to alcohols in the range of 3229–3400 cm^–1^, as well as the stretch ν­(C=C) attributed to the carboxymethyl group at 1679 cm^–1^, were observed. The stretch ν­(C–C) characteristic of the cycloolefin was also present at 1624 cm^–1^. In addition, the band located between 1105 and 1200 cm^–1^ corresponds to the ν­(C–O) bond of the ester/alcohol. In the MANAE-agarose and MANAE-agarose cross-linked with genipin spectra, stretches related to the alkene methylene group ν­(C–H) at 1370 cm^–1^ and the glycosidic bond ν­(C–O–C) at 1059 cm^–1^, associated with the agarose structure, were observed. For agarose and genipin functionalized with amino groups, a stretch was detected at 3077 cm^–1^, which was assigned to the primary amine ν­(N–H).^36,37^ The Schiff base (imine) was formed as a result of the bond between genipin and the amino groups of the MANAE-agarose support, as evidenced by the emergence of the stretch v­(C=N) at 1644 cm^–1^ in the MANAE-agarose cross-linked with genipin support spectrum (Figure 2b).^16,38^

**Figure 2:**
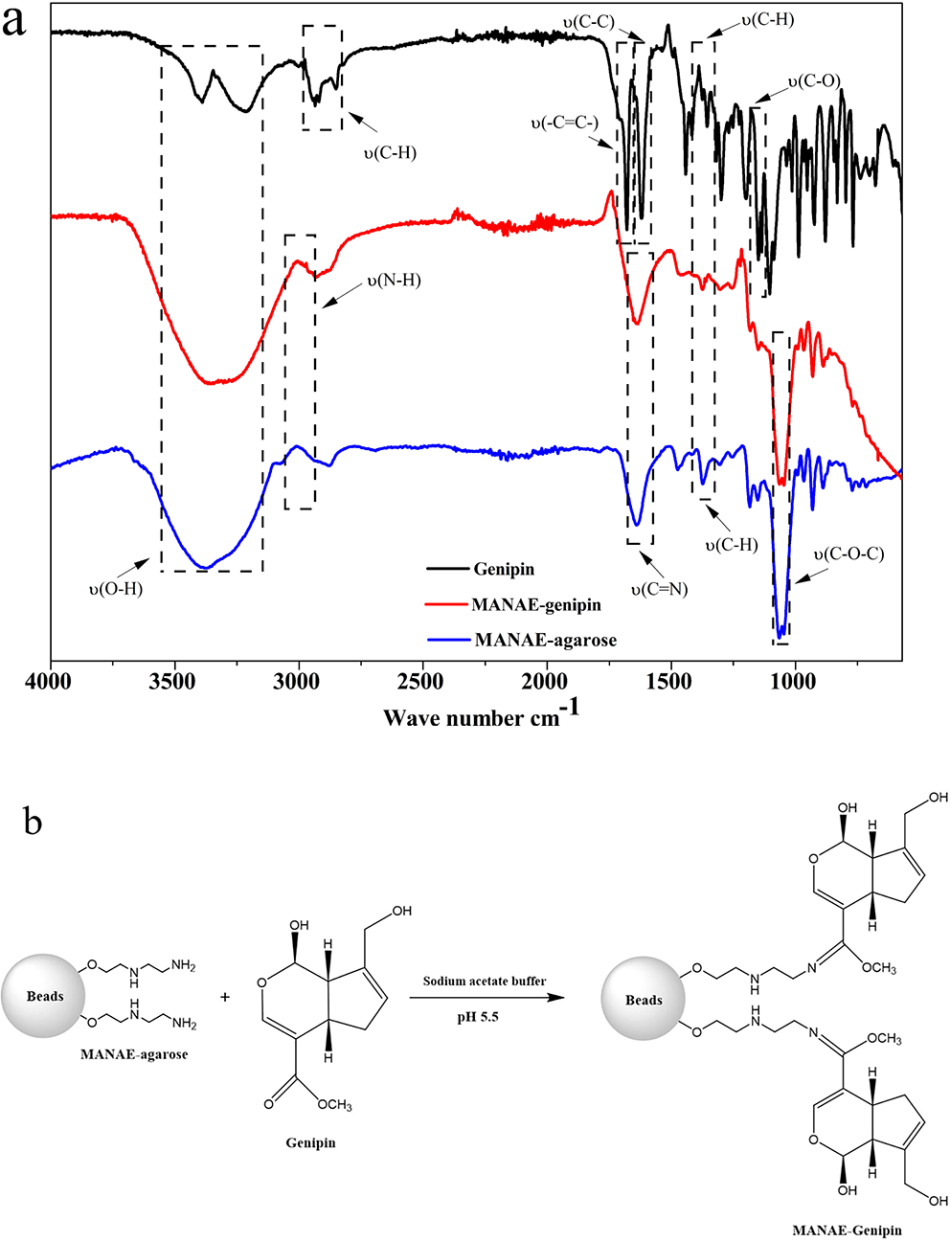
(a) Vibrational spectrum in the infrared region (FTIR) of the MANAE-agarose support (blue), MANAE-genipin support (red), and genipin (black). (b) Illustrated diagram of the reaction between MANAE-agarose and genipin to form MANAE-genipin.

### 3.3 Immobilization of FTase

Purified *A. aculeatus* OI4C2 FTase was efficiently immobilized on MANAE-genipin, resulting in an immobilization yield of 98.53%, recovered enzymatic activity of 76.71%, and overall immobilization efficiency of 77.86%. Comparatively, commercial fructosyltransferases from *A. aculeatus* have been immobilized on various supports, including Eupergit beads (96% immobilization yield),^39^ calcium alginate (50.7% yield),^40,40^ and magnetic Fe_3_O_4_–chitosan nanoparticles cross-linked with 3% glutaraldehyde (94.84% yield).^41^

The use of nontoxic support materials and cross-linking agents is critical for enzymes, such as FTase, particularly given their applications in the food and pharmaceutical industries. Genipin is a substance found in the fruit of the jenipapo (*G. americana* L.), a fruit native to Brazil^42^ known for its pharmacological properties.^23,43^ Genipin may be an excellent option for enzyme immobilization processes because it is safer than traditional cross-linking agents, such as glutaraldehyde, which is neurotoxic and teratogenic at high concentrations for food and pharmaceutical applications.^23,44^ The findings indicate that MANAE-agarose cross-linked with genipin offers an excellent combination of enzyme retention and safety for industrial applications as a support for FTase immobilization.

### 3.4 Comparative Biochemical Characterization of Free and Immobilized FTase

#### 3.4.1 Effect of Temperature

The optimal temperature for the activity of free and immobilized FTases exhibited a similar profile (Figure 3a). However, the immobilized enzyme showed slightly lower activity between temperatures of 20 and 60 °C compared to the free form, while displaying a 10 °C upward shift in the maximum activity, reaching its optimum at 70 °C (Figure 3a). A comparable behavior was reported for FTase from *A. flavus* NFCCI2364 immobilized in alginate beads, in which the optimum temperature increased from 50 °C (free enzyme) to 60 °C after immobilization.^45^ The reduced activity observed at moderate temperatures (20–60 °C) may be attributed to the reduced conformational flexibility imposed by multipoint covalent bonding, which can limit catalytic efficiency under mild conditions. In contrast, the enhanced rigidity provided by immobilization delays thermal unfolding, resulting in a 10 °C upward shift in the optimum temperature.^46–48^

**Figure 3:**
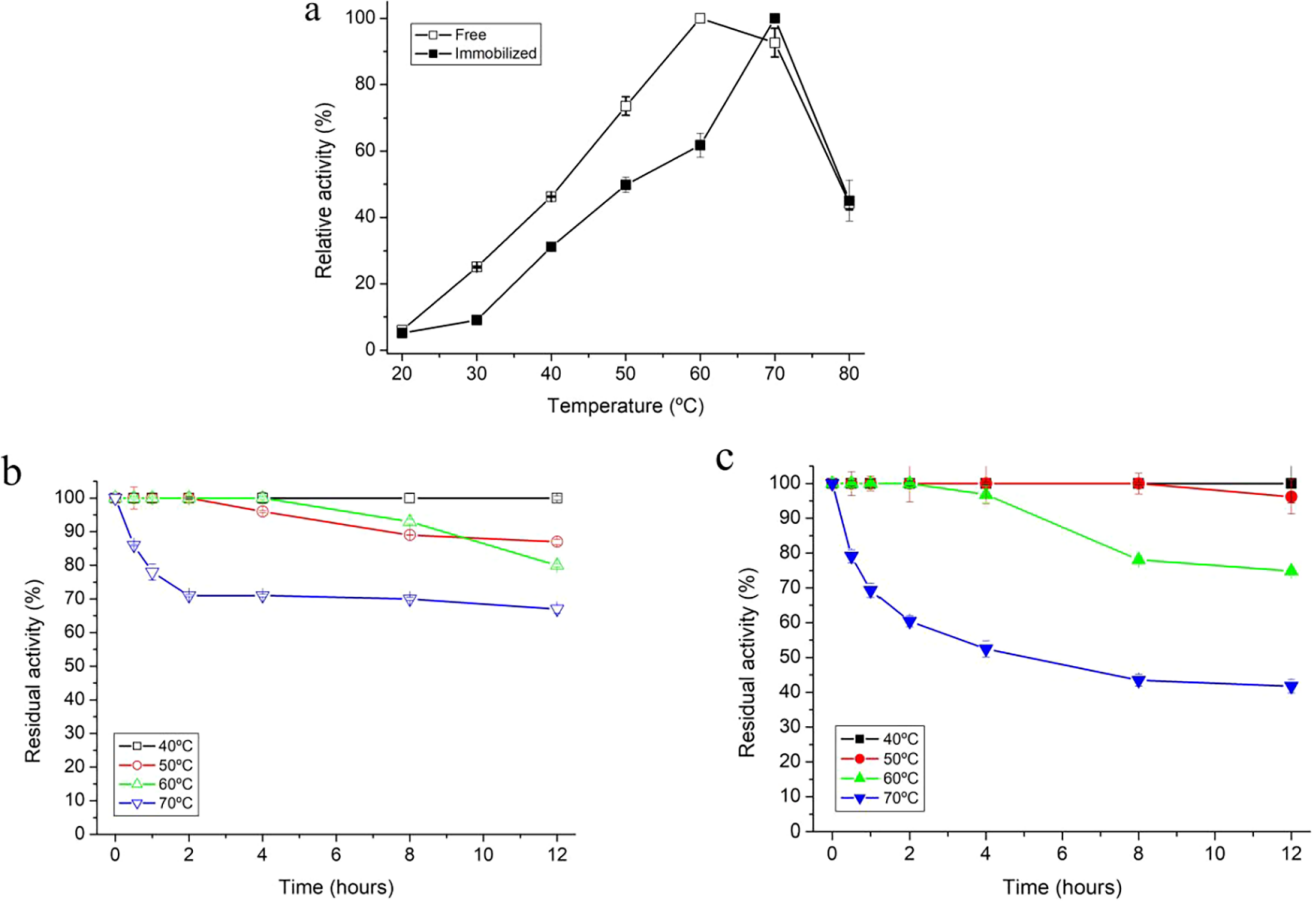
Effect of temperature on free and immobilized FTase activity (a); thermal stability of free (b) and immobilized (c) FTase at an incubation period of up to 12 h at temperatures of 40, 50, 60, and 70 °C.

Thermal stability was evaluated by measuring the residual activity (%) of free and immobilized FTases after incubation at temperatures of 40–70 °C for a period of 12 h (Figure 3b,c). Immobilized FTase exhibited greater stability at 40 and 50 °C over a 12 h period compared to the free enzyme (Figure 3b,c), indicating its ability to continuously produce FOS at moderate temperatures without undergoing significant enzymatic denaturation. Although the immobilized enzyme exhibited an optimum temperature of 70 °C, its half-life at this temperature decreased to approximately 4 h, indicating faster thermal inactivation under severe conditions. These results indicate that FTase immobilized in MANAE-agarose with genipin improves FTase stability at moderate temperatures while increasing susceptibility to denaturation at higher temperatures. This thermal stability of the immobilized enzyme may be due to the rigidification of the structure of the immobilized FTase by the multipoint covalent bond to the MANAE-agarose support cross-linked by genipin, which remains unchanged even at extreme temperatures.^46^ In addition, although the free FTase shows slightly higher activity at its optimum temperature, the MANAE–genipin immobilization strategy offers industrial advantages in terms of operational stability, extending processing time, and reusability, which directly reduce enzyme consumption and production costs.

#### 3.4.2 Effect of pH

The optimum pH for FTase activity was the same for both the free and immobilized enzymes at pH 5.5 (Figure 4a). FTase immobilized on MANAE-agarose cross-linked with genipin retained approximately 70% of its initial activity within the pH range of 4.5–6.5 after 72 h. The immobilized FTase showed markedly higher resistance to acidic pH (4.5 and 5.0) due to the reduced conformational flexibility and protection of catalytic residues provided by the MANAE–genipin matrix. This is a clear advantage over the native enzyme, which is inactivated under acidic conditions (Figure 4b,c). As many food-industry processes operate at low pH, this improved acid stability supports longer operation, reduced activity losses, and higher reusability, enhancing the applicability of immobilized FTase. This behavior suggests that immobilization provides a protective microenvironment, reducing enzyme denaturation under acidic conditions and thereby expanding the stability range.^49^ This protective effect has often been attributed to the microenvironment created by the support, which can mitigate structural destabilization at extreme pH values.^46,47^ In contrast, FTase from *A. flavus* NFCCI2364 immobilized on chitosan displayed higher stability at near-neutral to alkaline values (pH 6.5–8.0).^45^ Similarly, commercial FTase (Pectinex Ultra SP-L) immobilized on Fe_3_O_4_–chitosan magnetic nanoparticles exhibited an optimal pH of 7.0.^41^

**Figure 4:**
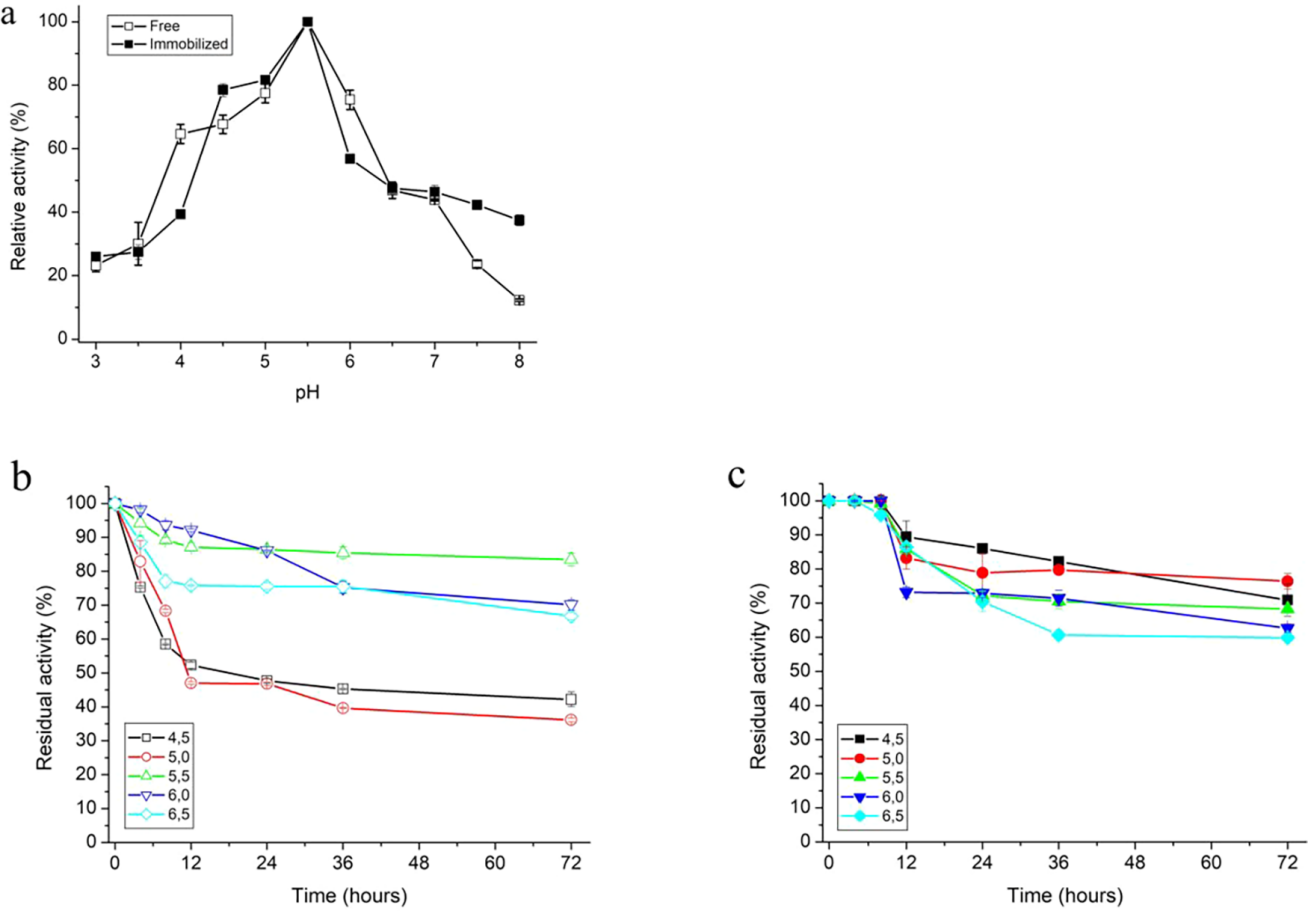
Effect of pH on free and immobilized FTase activity (a); pH stability of free (b) and immobilized FTase (c).

#### 3.4.3 Kinetic Parameters

The apparent *K*_M_ values of *A. aculeatus* FTase with sucrose were 0.82 and 0.94 M for the free and immobilized forms, respectively (Table 2). Because lower *K*_M_ values indicate greater substrate affinity, the free enzyme exhibited a higher affinity for sucrose than the immobilized form. The apparent *V*_max_ of the free enzyme (131,926 μmol min^–1^ mg^–1^ protein) was also considerably higher than that of the immobilized form (16,129 μmol min^–1^ mg^–1^ protein). The immobilized FTase showed a slightly higher apparent *K*_M_, indicating reduced apparent affinity toward sucrose, and a pronounced decrease in apparent *V*_max_. These differences suggest that immobilization may restrict mass transfer and reduce the flexibility of the catalytic site, thereby limiting the transfer of fructosyl groups and lowering the apparent catalytic efficiency.^50,51^

**Table 2:** Kinetic Parameters of Free and Immobilized *A. aculeatus* FTase Using Sucrose as a Substrate

enzyme form	*K*_M,app_ (M)	*V*_max,app_ (μmol min^–1^mg^–1^)
free FTase	0.82	131,926
immobilized FTase	0.94	16,129

Comparable results have been reported for other immobilized FTases. For instance, Tanriseven and Aslan^39^ observed increased *K*_M_ values upon immobilization of the commercial FTase from Pectinex Ultra SP-L on Eupergit C (47 g L^–1^ for the free enzyme versus 91 g L^–1^ for the immobilized form), while *V*_max_ values decreased only slightly (86 versus 82 g glucose L^–1^ min^–1^, respectively).

#### 3.4.4 Operational Stability

The FTase immobilized on MANAE-agarose cross-linked with genipin remained with high activity, retaining 86.06 and 83.49% of its initial activity after the second and third cycles of reaction, respectively. Remarkably, immobilized FTase maintained 48% residual activity even after 12 consecutive cycles (Figure 5). These results were considerably higher than those reported for *Saccharomyces cerevisiae* FTase immobilized on cobalt ferrite magnetic nanoparticles, which retained only 20% of its relative activity after 12 reuse cycles.^50^ Similarly, immobilization of *A. niger* FTase resulted in a 50% reduction in its initial activity within three cycles.^5^

**Figure 5:**
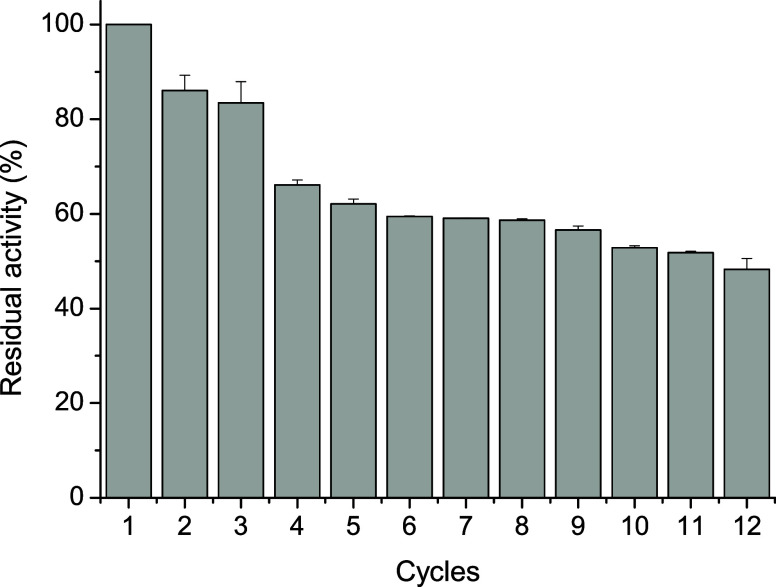
Operational stability of MANAE-agarose cross-linked with genipin-FTase.

The result of expanded reuse of *A. aculeatus* FTase immobilized on MANAE-agarose cross-linked with genipin from this study highlights its potential to substantially reduce operational costs and improve the economic viability of FOS production.^41^ Therefore, the immobilization method used in this study was effective in making the enzymes more reusable and showed that FTase immobilized on MANAE-agarose cross-linked with genipin could be used on a large scale to produce FOS.

### 3.5 FOS Synthesis

The FOS synthesis products by free and immobilized FTase were analyzed by HPLC (Figure 6a,b). The production of kestose was detected after 30 min of reaction, followed by nystose, which was generated after 12 h. The sucrose concentration decreased concomitantly throughout the reaction, while fructose levels remained insignificant, indicating high transfructosylation activity catalyzed by the enzyme. Immobilized FTase converted sucrose into 97.37 g L^–1^ of FOS (40.34%) after 36 h, which consisted of 80.17 g L^–1^ of kestose and 17.20 g L^–1^ of nystose. In comparison, free FTase produced only 73.44 g L^–1^ of FOS (27.04%) under the same conditions. Choukade and Kango^52^ reported 55.14% FOS yield after 48 h of incubation using mycelial FTase with 50% sucrose. Therefore, long reaction times with FTases can further improve the conversion of sucrose to FOS.

**Figure 6:**
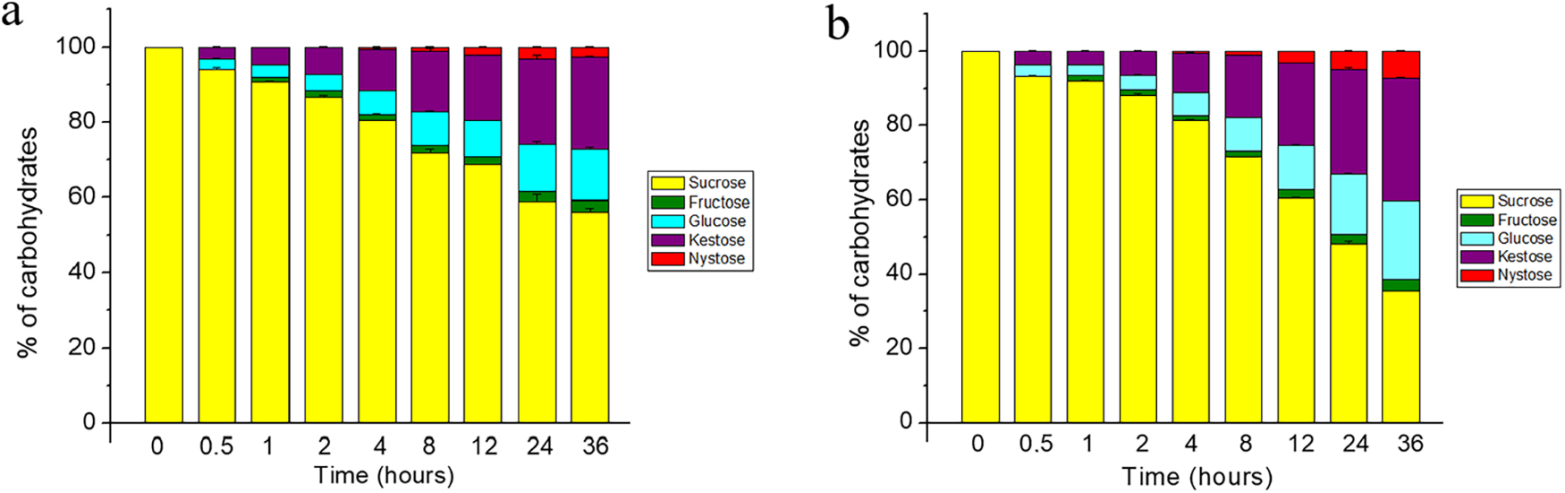
HPLC analysis of the products obtained from transfructosylation by free (a) and immobilized (b) FTase over a period of up to 36 h.

This study demonstrated that *A. aculeatus* OI4C2 fructosyltransferase can be efficiently immobilized on MANAE-agarose cross-linked with the natural cross-linking agent genipin, resulting in a stable, reusable biocatalyst to produce fructooligosaccharides. The immobilization strategy led to high immobilization yield and activity recovery, improved thermal and acidic pH stability, and significant operational stability over multiple reuse cycles. Importantly, the immobilized enzyme exhibited superior FOS production compared to the free form, favoring the synthesis of short-chain fructooligosaccharides, mainly kestose and nystose, which are of high interest for food and nutraceutical applications. The use of genipin as a nontoxic cross-linker highlights the sustainability and safety of the proposed immobilization system. Thus, the results provide a robust foundation for the application of MANAE–genipin-immobilized fructosyltransferase in industrial FOS production and support further process optimization and scale-up studies.

## Supplementary Material



## Data Availability

Data will be made available on request.

## Notes

Chromatographic profile of fructosyltransferase activity from *A. aculeatus* on DEAE-Sepharose column; Tris-HCl buffer (20 mM, pH 7.2); NaCl gradient elution. (●) Absorbance at 540 nm; (□) absorbance at 280 nm (Figure S1); chromatographic profile of fructosyltransferase activity from *A. aculeatus* on Sephadex G-75 column. (●) Absorbance at 540 nm; (□) absorbance at 280 nm (Figure S2) (PDF)
