# Supplementary material for: Innovative Immobilization of Aspergillus aculeatus OI4C2 Fructosyltransferase on MANAE-Agarose Cross-Linked with Genipin for Enhanced Production of Fructooligosaccharides
Source: J Agric Food Chem. 2026 Feb 23;74(8):6951–9. doi: 10.1021/acs.jafc.5c13809 (PMC12964536; doi:10.1021/acs.jafc.5c13809)
Supplement: Supplementary file 1 [file jf5c13809_si_001.pdf]

Supporting Information

Innovative immobilization of *Aspergillus aculeatus* OI4C2 fructosyltransferase in MANAE-agarose cross-linked with genipin enhanced fructooligossacharides production

Tatiane Sayuri Inagaki[a], Diandra de Andrades[b], Gabriela Souza Alves Picciarelli [b], Maria de Lourdes Teixeira de Moraes Polizeli[b], Douglas Poleto de Oliveira[c], Valdemiro Pereira de Carvalho Junior[c], Paula Daniela Helfenstein Rother[d], Mariana Fensterseifer Fabricio[d], Marco Antônio Záchia Ayub[d], Plinho Francisco Hertz[e], Marina Kimiko Kadowaki[a]*

[a]Center of Medical Sciences and Pharmaceutical, Western Paraná State University, Rua Universitária 2069, ZC 85819-110 Cascavel, PR, Brazil

[b]Department of Biology, Faculty of Philosophy, Sciences and Letters of Ribeirão Preto, University of São Paulo, Ribeirão Preto 14040-901, SP, Brazil

[c]Department of Chemistry and Biochemistry, Faculty of Science and Technology, Júlio de Mesquita Filho State University, Presidente Prudente 19060-900, SP, Brazil

[d]Biotechnology, Bioprocess, and Biocatalysis Group, Food Science and Technology Institute, Federal University of Rio Grande do Sul, Av. Bento Gonçalves 9500, PO Box 15090, ZC 91501-970 Porto Alegre, RS, Brazil

[e]Enzymology Laboratory, Food Science and Technology Institute, Federal University of Rio Grande do Sul, 9500 Bento Gonçalves Ave, P. O. Box 15090, ZC 91501-970 Porto Alegre, RS, Brazil

*Corresponding author: Marina K. Kadowaki (marinakk@gmail.com)

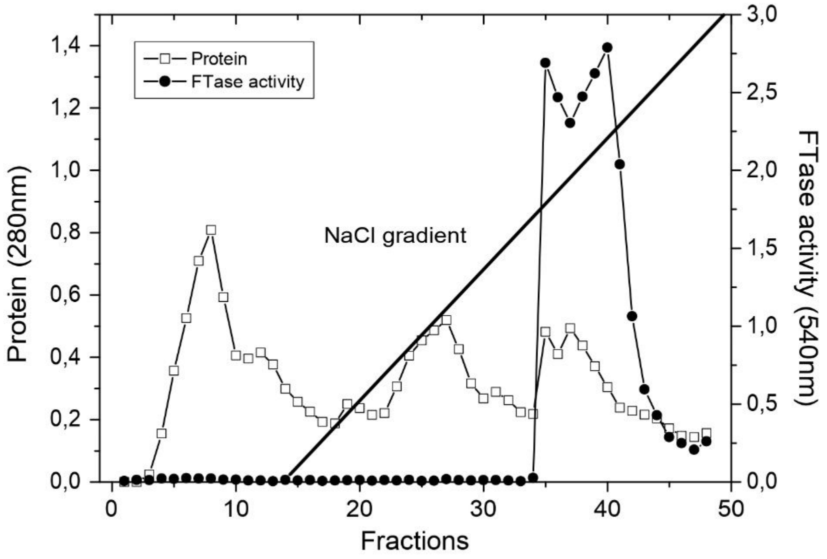

Figure S1: Chromatographic profile of fructosyltransferase activity from *A. aculeatus* using a DEAE-Sepharose column equilibrated with Tris-HCl buffer (20 mM, pH 7.2) and eluted using a NaCl gradient. (●) absorbance 540 nm; (□) absorbance 280 nm.

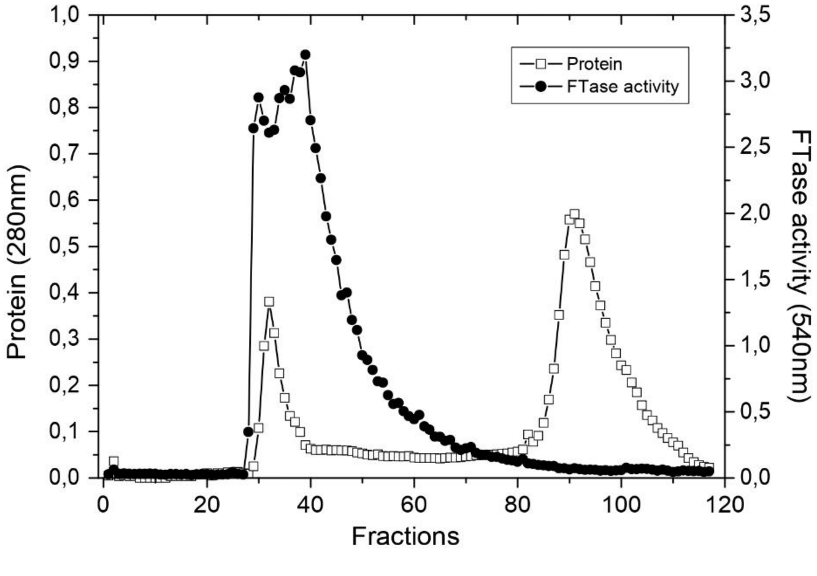

Figure S2: Chromatographic profile of fructosyltransferase activity from *A. aculeatus* using a Sephadex G-75 column, proteins were eluted with 20 mM sodium acetate buffer (pH 5.5). (●) absorbance 540 nm; (□) absorbance 280 nm.
